# Compositional Differences of Meconium Microbiomes of Preterm and Term Infants, and Infants That Developed Necrotizing Enterocolitis or Feeding Intolerance

**DOI:** 10.3390/pathogens12010055

**Published:** 2022-12-29

**Authors:** Hyun Mi Kang, Sol Kim, Seok Hwang-Bo, In Hyuk Yoo, Yu-Mi Seo, Moon Yeon Oh, Soo-Ah Im, Young-Ah Youn

**Affiliations:** 1 Department of Pediatrics, Seoul St. Mary’s Hospital, Catholic University, Seoul 06591, Republic of Korea; 2 Vaccine Bio Research Institute, College of Medicine, The Catholic University of Korea, Seoul 06591, Republic of Korea; 3 Department of Radiology, Seoul St. Mary’s Hospital, College of Medicine, The Catholic University of Korea, Seoul 06591, Republic of Korea

**Keywords:** meconium, microbiome, preterm

## Abstract

The primary aim of this study was to investigate the compositional differences of the first passed meconium microbiome in preterm and term infants, and the secondary aim was to compare the meconium microbiomes of preterm and term infants that later developed necrotizing enterocolitis (NEC)/Feeding intolerance (FI) compared to those that did not develop NEC/FI. During the study period, a total of 108 preterm and term newborns' first passed meconium occurring within 72 hours of birth were collected and microbiome analyzed. Meconium microbiomes showed a disruption in the percentages of the core microbiome constituents in both the phylum and genus levels in infants born < 30 weeks of gestational age (GA) compared to those born ≥ 30 weeks of GA. In the phylum level, *Bacteroidetes* and *Firmicutes,* and in the genus level, *Prevotella* and *Bacteroides,* were predominant, with *Prevotella* accounting for 20–30% of the relative abundance. As GA increased, a significant increase in the relative abundance of *Bacteroidetes* (*P* for trend < 0.001) and decrease in *Proteobacteria* (*P* for trend = 0.049) was observed in the phylum level. In the genus level, as GA increased, *Prevotella* (*P* for trend < 0.001) and *Bacteroides* (*P* for trend = 0.002) increased significantly, whereas *Enterococcus* (*P* for trend = 0.020) decreased. Compared to the control group, the meconium of infants that later developed NEC/FI had significantly lower alpha diversities but similar beta-diversities. Furthermore, the NEC/FI group showed a significantly lower abundance of *Bacteroidetes* (*P* < 0.001), and higher abundance of *Firmicutes* (*P* = 0.034). To conclude, differences were observed in the composition of the first passed meconium in preterm and term infants that later develop NEC/FI compared to those that did not.

## 1. Introduction

In the last few decades, improvements in the survival rate of preterm infants have been observed [1]. The management and prevention of complications arising from preterm birth and immature organs, such as necrotizing enterocolitis (NEC), feeding intolerance (FI) and sepsis, are crucial to the developmental outcome of these infants. Prematurity is a consistent independent risk factor of NEC, and NEC incidence is known to increase with decreasing birth weight and gestational age (GA) [2,3]. However, prematurity itself cannot entirely explain the pathogenesis of NEC/FI because some infants born at higher GA may also manifest NEC/FI. Due to the multifactorial and complex etiology, the pathogenesis of NEC has remained elusive and controversial until now [4,5,6]. 

The intestinal microbiota performs several functions, including maturation of the immune system, regulating immune response, protection against the invasion of opportunistic pathogens, and mediating hormonal regulation [7]. Prior to birth, infants are isolated from exposure to microorganisms in the environment [8], and fetal meconium has been reported to be without any detectable microbiota before birth [9]. Other studies suggest that the environment in utero is not sterile, and therefore the first passed meconium may somewhat mirror the in utero microbial environment [10,11]. Nevertheless, perinatal exposures to microbes lead to transient or long-term colonization. Therefore, perinatal events such as the method of delivery affect the types of microbes that infants are exposed to, resulting in an establishment of different types of gut microbiomes [12,13,14].

The preterm gut microbiome undergoes early disruption before achieving bacterial maturation. Gestational age at birth, representing the timing of first microbial exposure, is known to be significantly associated with the microbial composition including biomass and diversity of meconium in preterm infants [14,15]. In fact, according to a study examining the relationship of NEC to GA, events occurring at or soon after birth was found to be important in NEC development [16]. The development of NEC highly involves gut mucosal immunity, comprising intestinal cell signaling and gut microbiota [17]. The immature intestine of premature infants may be in a hyperactive state with increased inflammatory responses and impairment of intestinal perfusion [18]. Thus, the timing and state of the preterm infants’ gut can influence the composition of the gut microbiome leading to the development of NEC. 

In order to understand how the composition of the gut microbiome in the immediate perinatal period may affect the clinical course of preterm and term infants, the primary aim of this study was to investigate the composition of the first passed meconium microbiome in Korean preterm and term infants. The secondary aim was to compare the meconium microbiomes of preterm and term infants that later developed NEC/FI versus those that did not develop NEC/FI.

## 2. Materials and Methods

### 2.1. Study Population and Sample Collection

This was a prospective study carried out at a neonatal intensive care unit (NICU) of a tertiary referral university hospital located in Seoul, Korea. Preterm and term infants admitted to the NICU during May 2021 to February 2022 were included as study participants. The inclusion criteria were as follows: (1) first meconium stool sample obtained within 72 hours of birth during the study period, (2) admitted to the NICU regardless of the GA at birth, (3) transferred to the NICU immediately after birth for any symptoms requiring intensive care, and (4) obtained informed consent from the legal guardians. Exclusion criteria were as follows: (1) transferred to the NICU after being admitted to the nursery or another hospital, and (2) first meconium stool passed 72 hours or more after birth. 

Immediately upon passage, 1–2 mL (maximum 3 mL) of the infant’s first passed meconium was collected and stored at −20 °C. The specimens were then transferred to a deep freezer set at −80 °C until DNA was extracted for microbiome analyses. The clinical course and progression of the infants included in the study were surveyed until death or discharge. Demographic and clinical data, including GA, birth weight, history of sepsis, and postnatal hospital outcomes were obtained. The patients were divided into two groups: NEC/FI group comprised 26 (24.1%) infants that were diagnosed with NEC/FI during the course of prospective monitoring, and the control group consisting of 82 infants (75.9%) that did not present with any GI (gastrointestinal) symptoms during the NICU admission period. 

The study was approved by the Ethics Committee of Seoul St. Mary’s Hospital, The Catholic University of Seoul, Korea (IRB no. KC21TISI0329). All legal guardians of the study participants gave approval and signed informed consent forms for study participation. 

### 2.2. Fecal Microbiome Analysis; DNA Extraction and Amplification

DNA was extracted using TIANLONG ®-nucleic acid extraction kit (for stool DNA/RNA extraction) (TianLong Science and Technology Co., Xi’an, China) then determined concentration and purity using nanodrop spectrophotometer. The target region (V3-V4) was amplified using PCRBIO VeriFi Mix (PCR Biosystems®, London, UK) and 16S-amplicon primer (Macrogen®, Seoul, Republic of Korea) at 95 °C for 3 min hot start followed by 25 cycles of 95 °C for 30 s and 55 °C for 30 s, 72 °C for 30 s with a final elongation step of 72 °C for 5 min. The V3-V4 target region reverse primer (5'TCGTCGGCAGCGTCAGATGTGTATAAGAGACAGCCTACGGGNGGCW-GCAG3’) and forward primer (5'GTCTCGTGGGCTCGGAGATGTGTATAAGAGACAGGACT-ACHVGGGTATCTAATCC3’) were used according to the guideline ‘Preparing 16S Ribosomal RNA Gene Amplicons for the Illumina MiSeq System’ [19].

The amplified DNA subsequently were purified automatically in Nucleic Acid Extractor (TianLong Science and Technology Co.) using MagListo™ PCR/Gel Purification Kit (Bioneer®, Daejon, Republic of Korea), then the size and concentration were determined using Qsep100 (Bioptic®, Changzhou City, China). Purified samples were then standardized to 8 ng per reaction (total reaction volume of 20 µL) and Index PCR was performed to attach in dual indices and Illumina sequencing using PCRBIO VeriFi Mix (PCR Biosystems®, London, UK) and Nextera ® Index kit V2 Set A (Illumina®, San Diego, CA, USA) at 95 °C for 3 min hotstart followed by 8 cycles of 95 °C for 30s and 55 °C for 30 s, 72 °C for 30 s with a final elongation step of 72 °C for 5 min. 

After indexing, the final library was cleaned up using MagListo™ PCR/Gel Purification Kit (Bioneer®) before quantification. DNA size and concentration were again measured using Qsep100 (Bioptic®) and Qbit flex fluorometer (Invitrogen®). After Qbit and Qsep measurements were completed, the mixing volume value was calculated then the PCR product was pooled into a microtube. After mixing, the final pooled library concentration and size were checked using Qbit and Qsep. The counts per reaction for each microbiome species was recorded. 

### 2.3. Sequencing and Data Analysis

Libraries were prepared using MiSeq ® Reagent Kit V3 600 cycles kit (Illumina®), pooled libraries were denatured with NaOH 0.2 N, then diluted with hybridization buffer before sequencing, PhiX was used as an internal control for each run. DNA were pooled and sequenced on an Illumina Miseq platform according to the manufacturer’s standard instruction, sequencing data of bacterial variability sites (V3-V4) were analyzed with 16S metagenomics app. Analysis was performed on Quantitative Insights into Microbial Ecology 2 (QIIME2; version 2020.2 and 2020.6) [20]. DNA reads under 200 bp were omitted from the taxonomic analysis. Reads were then demultiplexed and denoised with DADA2 [21]. Denoised reads were trimmed at 15 and truncated at 260, and chimeric reads were filtered out, resulting in a total of 3,994,640 processed reads ready for further analyses. R package decontam (version 1.8.0) was used to filter out environmental contaminants from each sample type [22].

### 2.4. Definitions

NEC was defined as stage II or above, according to the modified Bell’s staging classification grade [23], which includes one or more of the following clinical signs: bilious, gastric aspirator emesis, abdominal distention, or occult or gross blood in stool. This classification also includes one or more of the following radiographic findings: pneumatosis intestinalis, hepatobiliary gas, or pneumoperitoneum. Therapeutic decisions were based on clinical staging. FI was defined as persistent gastric aspirates of >50% of the feed volume with or without increased abdominal girth in the absence of culture-positive sepsis or radiographic evidence of NEC for 48 hours [24], more than 3 times a day which did not allow the advancement of feeding > 10–20 mL/kg/day. Respiratory distress syndrome (RDS) was diagnosed based on both clinical and radiographic findings. Bronchopulmonary dysplasia was defined as use of oxygen ≥ 0.21 at 36 weeks.

Preterm infants were defined as infants born < 37 weeks of gestational age, late preterm as GA 34 to <37 weeks, moderately preterm as GA 32 to <34 weeks, very preterm as GA 28 to <32 weeks, and extremely preterm as GA < 28 weeks [25]. Small for gestational age was defined as a neonate born with birthweights below the <10th percentile for gestational age.

### 2.5. Statistical Analysis

For descriptive statistics with demographic data and concentration and continuous variables were presented as means and standard deviation (SD) while categorical variables were presented as percentages and frequencies. For inferential statistics, continuous variables were compared using the *t* test, one-way ANOVA or Wilcoxon rank sum test depending on the normality of the variable being tested. More specifically, we compared the microbiome concentrations in the NEC group versus controls using the student’s *t* test (or Welch–Satterthwaite *t* test when the variance in the two groups was unequal). The logistic regression analysis was used to find the *P* for trend. The alpha diversity was calculated using Shannon’s diversity index, and beta diversity was plotted using Principal Coordinate Analysis of Bray–Curtis dissimilarity. Differences in categorical variables are compared using the chi-square or Fisher’s exact test. A univariate and multivariate analyses was carried out on all possible perinatal factors associated with NEC/FI. A *p* value of <0.05 was considered statistically significant.

## 3. Results

### 3.1. Clinical Characteristics

During the one-year study period, a total 108 preterm and term infants born between GA 22 weeks to 40 weeks were included in the study. The mean GA was 34.7 (standard deviation [SD] ± 4.2) and mean birth weight was 2,305.9g (SD ± 863.0). A total 83.3% (n = 90/108) of the patients were born via caesarean section, and 88.9% (n = 96/108) were male (Table 1). A total 58.3% (n = 63/108) were preterm infants with a mean GA 31.6 weeks (SD ± 3.7) and birth weight 1,807.4 (SD ± 773.0) g. A total 34.9% (n = 22/63) of preterm infants and 8.9% (n = 4/45) term infants were diagnosed with NEC/FI (*P* = 0.003) (Table 1). 

### 3.2. Distribution of Microbiota according to Gestational Age

**Figure 1:**
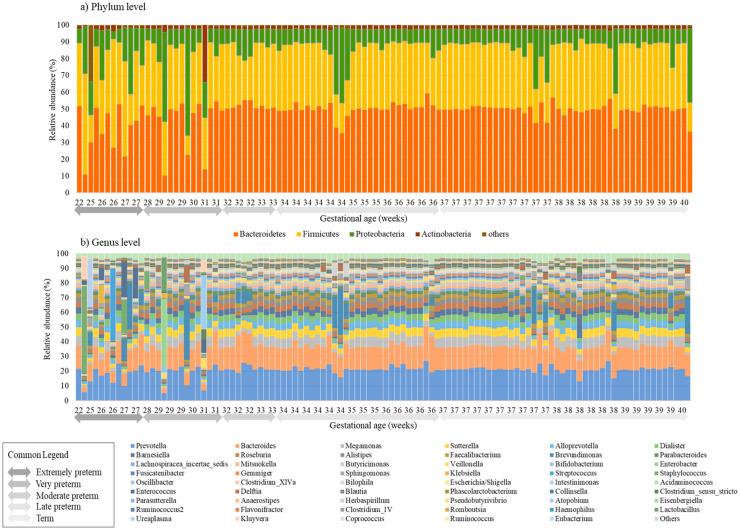
**Distribution of** (**a**) **phylum levels and** (**b**) **genus levels according to gestational age.** Microbiomes of the 1st stool samples obtained after delivery showed dysbiosis in extremely preterm babies born < 31 weeks of gestational age in both the phylum and genus levels. In the phylum level, *Bacteroidetes* and *Firmicutes* were the most dominant phyla in the first neonatal microbiomes. In the genus level, *Prevotella* sp. and *Bacteroides* sp. were the most predominant species in the meconium.

Compared to the meconium microbiomes of infants born after the 30th week of gestation, those born prior to 30 weeks showed a disruption in the percentages of the core microbiome in both the phylum and genus levels (Figure 1). *Bacteroidetes* and *Firmicutes* were dominant in the first meconium microbiomes (Figure 1a), and in the genus level, *Prevotella* and *Bacteroides* were the most predominant species, with *Prevotella*’s relative abundance accounting for 20–30% of the gut microbiome. Furthermore, *Bacteriodes* composed the next large proportion, of up to ~20% of the microbiome. *Bifidobacterium* was not significantly different according to GA (Figure 1b).

**Figure 2:**
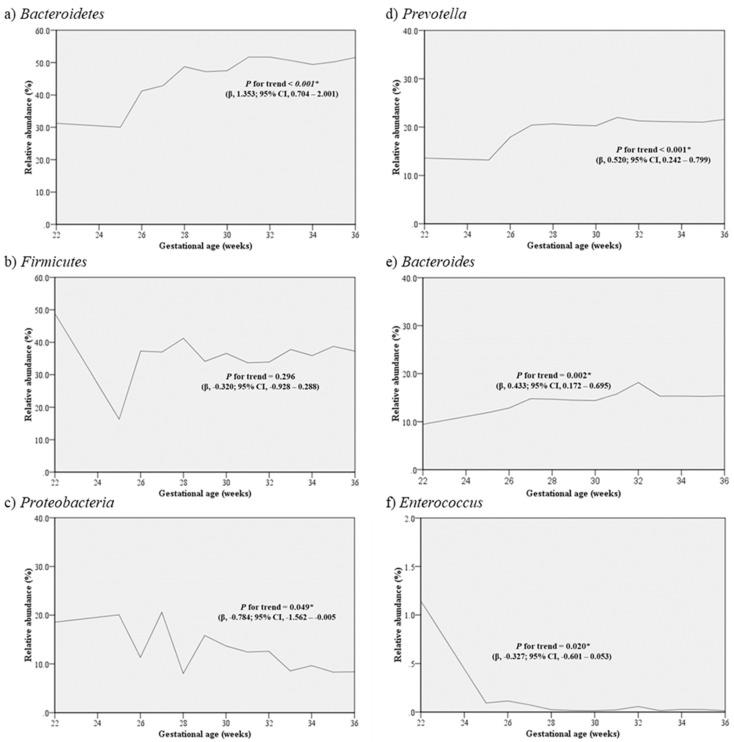
Change in the relative abundance of the phyla (**a**) *Bacteroidetes*, (**b**) *Firmicutes*, (**c**) *Proteobacteria* and genus (**d**) *Prevotella*, (**e**) *Bacteroides*, (**f**) *Enterococcus* according to gestational age in preterm infant born prior to 37 weeks of gestation (n = 63). The graph shows the median relative abundance for each gestational age.

In an analysis including only preterm infants born prior to 37 weeks of GA (n = 63), an analysis of the relationship between GA and relative abundance of the phyla *Bacteroidetes* in the meconium increased significantly as GA increased (*P* for trend < 0.001), and *Proteobacteria* decreased significantly as GA increased (*P* for trend = 0.049). In the genus level, as gestational age increased, *Prevotella* (*P* for trend < 0.001) and *Bacteroides* (*P* for trend = 0.002) increased significantly as GA increased, whereas *Enterococcus* (*P* for trend = 0.020) decreased as GA increased (Figure 2). 

### 3.3. Comparison of Microbiomes and Clinical Outcomes between NEC/FI Group Versus Control Group

A total 24.1% (n = 26) were diagnosed with NEC or FI. Of these, 22 were preterm infants, and 4 were term infants. All possible perinatal and postnatal clinical factors associated with an increased risk for NEC/FI were investigated [26]. In the univariate analysis testing a total of 21 factors, GA (*P* < 0.001), birthweight (*P* < 0.001), antenatal steroid use (*P* = 0.003), congenital infection (*P* = 0.010), RDS (*P* = 0.002), and intraventricular hemorrhage (IVH) above grade II (*P* = 0.021) were factors associated with NEC/FI. However, in the multivariate analysis, after adjusting for confounding, none of these factors were independently significantly associated with NEC/FI in this cohort of patients (Table 2). 

**Figure 3:**
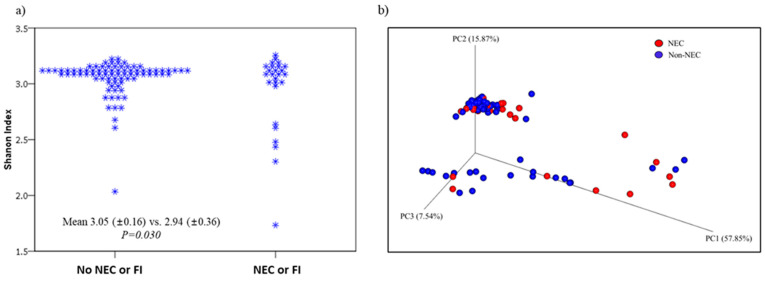
**Comparison of alpha diversities (Shannon Index) and beta diversities (Bray–Curtis dissimilarity)** of infants without any gastrointestinal symptoms versus those with NEC or FI. (**a**) Compared to the control group, infants that had NEC/FI had significantly lower alpha diversities however, (**b**) no significant differences in the beta diversities.

Compared to the control group, infants that had NEC/FI had significantly lower alpha diversities (Shannon Index, 3.05 vs. 2.94; *P* = 0.030, respectively), however, similar beta diversities (Figure 3). 

**Figure 4:**
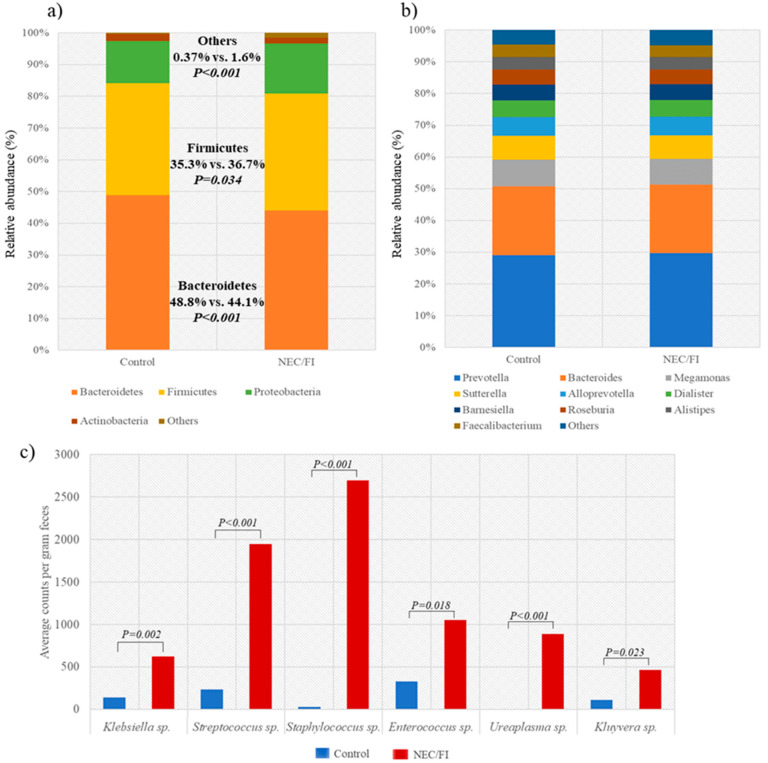
**Gut microbiome comparison between the control group versus NEC/FI group** of (**a**) absolute abundance and types of bacteria in the phylum level between the control group and NEC/FI group showing a significantly lower abundance of *Bacteroidetes*, and higher abundance of *Firmicutes* and others. (**b**) In the genus level, there were no significant differences in the absolute abundance of the 10 most dominant microbiota in the genus level, however, (**c**) the average counts in the NEC/FI group for the following species were significantly higher: *Klebsiella*, *Streptococcus*, *Staphylococcus*, *Enterococcus*, *Ureaplasma*, and *Kluyvera*.

The relative abundance of microbiota in the phylum level of the NEC/FI group showed a significantly lower abundance of *Bacteroidetes*, and higher abundance of *Firmicutes* and others (Figure 4a). In the genus level, there were no significant differences in the relative abundance of the 10 most dominant microbiota in the genus level (Figure 4b), however, the average counts in the NEC/FI group for the following species were significantly higher compared to the control group: *Klebsiella*, 624.6 [SD ± 2853.6] vs. 140.8 [SD ± 328.6], *P* = 0.002; *Streptococcus*, 1947.9 [SD ± 8891.1] vs. 237.8 [SD ± 152.8], *P* < 0.001; *Staphylococcus*, 2695.6 [SD ± 8790.8] vs. 26.8 [SD ± 95.0], *P* < 0.001; *Enterococcus*, 1053.4 [SD ± 3486.8] vs. 329.7 [SD ± 2052.6], *P* = 0.018; *Ureaplasma*, 886.5 [SD ± 4517.4] vs. 1.72 [SD ± 11.8], *P* < 0.001; *Kluyvera*, 462.1 [SD ± 2291.9] vs. 109.1 [SD ± 902.5], *P* = 0.023; respectively) (Figure 4c).

## 4. Discussion

The gut microbiota is a group of microorganisms, mainly bacteria, yeast, fungi, bacteriophages, and other viruses, as well as protozoa and archaea, which form a complex ecosystem in the human GI tract [27,28,29]. Meconium is often defined as the first stool passed within 48 h from birth. Neonatal microbiota starts diversifying quickly after birth, and compared to adults or older children, infant microbiota is known to have lower diversity as well as an unstable and highly dynamic microbiota structure [30]. This present study investigated the gut microbiome of 108 preterm and term infants' first passed meconium. The microbiomes of the meconium of preterm infants up to 30 weeks of GA showed lower alpha diversities and disruption in the percentages of the core microbiome in both the phylum and genus level, compared to infants born at a higher GA portraying a difference in the perinatal gut microbiome of preterm and term infants. 

The two phyla, *Bacteroidetes* and *Firmicutes*, constituted majority of the microbiota of the neonatal meconium, and as the GA increased, a significant increase in the relative abundance of *Bacteroidetes* and decrease in *Proteobacteria* was observed. A study from Germany also showed that GA was significantly associated with the composition of meconium from very preterm infants, and the most abundant phyla included *Firmicutes, Bacteriodetes, Proteobacteria*, and *Actinobacteria* [14]. Our study showed that in the genus level, *Prevotella* and *Bacteroides* were the predominant species, with *Prevotella*’s relative abundance accounting for 20-30% of the gut microbiome. Whereas in healthy adults, the two main phyla—*Firmicutes* and *Bacteroidetes*—constitute over 90% of the gut microbiota, followed by *Actinobacteria* and *Proteobacteria* [31,32]. 

Upon delivery, infants are exposed for the first time to a variety of microbes from various sources which lead to an establishment of the infants’ gut microbiome. Recent studies have shown that the predominant early colonizers of infant’s gut are maternal fecal bacteria, mainly *Bifidobacterium* and *Bacteroides*, and *Clostridium* [27,33,34,35]. Studies have also demonstrated that *Bacteroides* are associated with increased gut diversity and faster intestine maturation and natural childbirth has been shown to be significantly related to microorganisms reflecting the mother’s vaginal flora, such as *Bacteroides*, *Lactobacillus*, and *Prevotella* [36,37]. In the first passed microbiome of neonates in our study, *Prevotella* and *Bacteroides* were the most prevalent, accounting for about 50% of the core microbiome. However, because 83.3% (n = 90) were born via caesarean section, *Lactobacillus* was not a predominant genus in the meconium of the infants in our study. 

One of the most devastating gastrointestinal emergencies and a major cause of mortality in very low birth weight infants (VLBWI) is NEC. Furthermore, FI is also a frequently faced problem in many neonatal intensive care units (NICUs). Prenatal and postnatal factors, such as placental insufficiency, chorioamnionitis, gut ischemia, altered bacterial colonization, viruses, and blood transfusions, presumably disrupt the mucosal barrier, which may trigger inflammatory reactions in the immature intestines of preterm infants [4]. This study investigated whether a difference can be observed in the microbiome of meconium of infants that are eventually diagnosed with NEC. This study found that compared to infants that are discharged from the NICU without any gastrointestinal symptoms, infants that were diagnosed with NEC/FI had significantly lower alpha diversities in their first meconium. Furthermore, a significantly lower abundance of *Bacteroidetes*, and higher abundance of *Firmicutes* and other microbes were observed. *Firmicutes*, *Streptococcaceae*, and *Enterococcaceae* are known to predominate in adults with lower diversity gut microbiota [38]. Lower diversity gut microbiota is also known to be drivers of many diseases such as inflammatory bowel disease, acute diarrheal disease, C. difficile infection, and observed in cancer patients [38,39,40,41]. This shows that infants with NEC/FI have lower diversity dysbiosis, and this may have an important role for triggering inflammation leading to NEC/FI. 

In the study on meconium microbiome and its relation to neonatal growth and head circumference catch-up in preterm infants, *Polynucleobacter*, *Gp1*, and *Prevotella* appeared in greater abundance in meconium of preterm infants with adequate birth weight for GA [42]. In our study, we found that the composition of *Prevotella* was positively correlated with GA, suggesting that the abundance of *Prevotella* has an important role in the growth and development of preterm infants. 

The recent study conducted by Liu et al. on early gut microbiota in very low and extremely low birth weight preterm infants with feeding intolerance showed that the meconium samples of the FI group had higher proportions of γ-proteobacteria and *Escherichia-Shigella* and a lower proportion of *Bacteroides* compared with the those that did not have FI [43]. Although this was not corroborated in our study, our study found a lower proportion of *Bacteroides* in preterm infants with lower GA, suggesting that *Bacteroides* is a part of the healthy gut microbiome. Perturbation of the gut microbiota may promote overgrowth of pathobionts increasing the risk of infections and inflammation in the gut [44]. In a study on infants born via caesarean section, a high-level colonization by opportunistic pathogens was observed, including *Enterococcus*, *Enterobacter*, and *Klebsiella* [12]. In this study, the average counts in the NEC/FI group for the following pathobionts were significantly higher: *Klebsiella*, *Streptococcus*, *Staphylococcus*, *Enterococcus*, and *Ureaplasma*, and *Kluyvera.* The first moments of life are known to contribute to the formation of an NEC-associated microbiota [45], however, our study shows that the initiation in the formation of an NEC associated microbiota may even occur prior to events following birth.

In this study, there were six cases of placental abruption and 14 cases of PROM, meaning that these infants may have been exposed to the vaginal microbiota in utero, and subsequently affecting the results of the gut microbiome in these subjects. Due to the small number of subjects, further analyses were difficult. However, a study on 8 preterm neonates in Indonesia reported that PROM and mother’s diet influenced the meconium microbiome [46]. Further studies including a larger cohort of preterm infants are needed to make any conclusions on the impact of early exposure to vaginal microbiomes in utero and colonization and establishment of the infants’ gut microbiome. 

When compared with bacteria, the healthy human gut mycobiome is lower in biomass compared to bacteriome biomass. However, the roles of mycobiomes are increasingly recognized as important, whether it be beneficial or harmful. The gut mycobiome is known to be dominated by *Saccharomyces*, *Malassezia*, and *Candida* [47], and these pathobionts may potentially be harmful to preterm infants [48]. Further studies are needed to characterize the composition of mycobiomes in preterm infants to better understand its role in the immature gut. 

This study had a few limitations the sample size of the study groups was relatively small. However, to date, this study contains one of the largest cohort of neonates, from GA 22 to 40. Secondly, although not conclusive, studies have shown evidence of sex related difference in the gut microbiome [49]. Other studies do not support this [50]. In our study, 88.9% (n = 96) patient were male, therefore there may have been selection bias genders-wise. There is a lack of studies on the influence that gender has in the gut microbial composition of infants, and further studies are warranted. Finally, this study lacked a longitudinal analysis of changes in the gut microbiome of neonates after birth. However, the focus of our study was investigating the microbiome of the first meconium passed by the infant immediately after birth reflecting the perinatal gut microbiome of the infant. 

In summary, this study showed that low diversity dysbiosis was observed in the initial gut microbiomes of infants born premature, below 30 weeks of GA, and an increasing abundance of *Bacteroidetes* was observed as gestation age increased. Infants with NEC/FI had significantly lower alpha diversities, and a significantly higher counts of pathobionts such as *Klebsiella*, *Streptococcus*, *Staphylococcus*, *Enterococcus*, *Ureaplasma*, and *Kluyvera* were observed. To conclude, differences were observed in the composition of the first passed meconium in preterm and term infants that later developed NEC/FI compared to those that did not. These findings fuel the need to understand disease pathogenesis and develop novel and significant microbiota for earlier therapeutic and preventative strategies for vulnerable preterm babies in further research.

## Figures and Tables

**Table 1:** Comparison of perinatal characteristics of preterm and term infants included in the study.

	No. of Participants (%)			*P* Value
	Total N = 108	Preterm N = 63	Term N = 45	
GA, weeks (mean ± SD)	34.7 ± 4.2	31.6 ± 3.7	37.9 ± 1.0	*<0.001*
Birth weight, g (mean ± SD)	2,305.9 ± 863.0	1,807.4 ± 773.0	3,003.8 ± 339.3	*<0.001*
Mother DM	11 (10.2)	8 (12.7)	3 (6.7)	*0.354*
IVF	6 (5.6)	5 (7.9)	1 (2.2)	*0.397*
Male	96 (88.9)	60 (95.2)	36 (80.0)	*0.026*
Caesarean section	90 (83.3)	58 (92.1)	32 (71.1)	*0.007*
Small for gestational age	6 (5.6)	3 (4.8)	3 (6.7)	*0.692*
Chorioamnionitis	2 (1.9)	2 (3.2)	0	*0.509*
PROM, hrs	14 (13.0)	11 (17.5)	2 (4.4)	*0.069*
Placental abruption	6 (5.6)	6 (9.5)	0	*0.040*
Oligohydramnios	10 (9.3)	10 (15.9)	0	*0.005*
Antenatal steroid use	43 (39.8)	43 (68.3)	0	*<0.001*
Congenital infection	7 (6.5)	7 (11.1)	0	*0.040*
Need for resuscitation at birth	50 (46.3)	40 (63.5)	10 (22.2)	*<0.001*
NEC/FI	26 (24.1)	22 (34.9)	4 (8.9)	*0.003*

Late preterm, n = 28; moderately preterm, n = 10; very preterm, n = 13; extremely preterm, n = 12; *Abbreviations*: DM, diabetes mellitus; FI, feeding intolerance; GA, gestational age; IVF, in vivo fertilization; NEC, necrotizing enterocolitis; PROM, preterm prolonged rupture of membranes.

**Table 2:** Factors associated with an increased risk for NEC/FI in preterm and term infants.

	Univariate		*P* Value	Multivariate		*P* Value
	OR	95% CI		OR	95% CI	
GA, weeks (mean ± SD)	0.716	0.624–0.820	*<0.001*	0.736	0.491–1.104	*0.138*
Birth weight, g (mean ± SD)	0.998	0.998–0.999	*<0.001*	0.999	0.997–1.001	*0.162*
Caesarean section	1.716	0.455–6.470	*0.425*			
PROM	3.214	0.971–10.638	*0.056*			
Preeclampsia	0.893	0.174–4.590	*0.892*			
Placental abruption	3.435	0.649–18.179	*0.147*			
Oligohydramnios	3.667	0.970–13.866	*0.056*			
IUGR	3.435	0.649–18.179	*0.147*			
Chorioamnionitis	-	-	*0.999*			
Antenatal steroid use	4.068	1.602–10.335	*0.003*	0.376	0.074–1.913	*0.239*
Congenital infection	9.524	1.725–52.593	*0.010*	1.275	0.179–9.097	*0.808*
RDS	4.227	1.673–10.682	*0.002*	0.207	0.033–1.309	*0.094*
Massive pulmonary hemorrhage	-	-	*0.999*			
Air leak	1.283	0.234–7.043	*0.774*			
Sepsis	-	-	*0.998*			
IVH > grade II	2.187	1.126–4.247	*0.021*	0.595	0.227–1.560	*0.291*
PDA	2.194	0.346–13.909	*0.404*			
CLD	-	-	*0.998*			
PAH	3.333	0.446–24.936	0.241			
No breast milk given enterally prior to NEC/FI	0.406	0.147–1.119	*0.081*			
Antibiotics use prior to NEC/FI	-	-	*0.999*			

*Abbreviations*: CI, confidence interval; CLD, chronic lung disease; GA, gestational age; IUGR, intrauterine growth restriction; IVH, intraventricular hemorrhage; NEC/FI, necrotizing enterocolitis/feeding intolerance; PAH, pulmonary artery hypertension; PDA, patent ductus arteriosus; Covariates used in the multivariate analyses were as follows: Gestational age, birth weight, antenatal steroid use, congenital infection, RDS, IVH grade III and IV.

## Data Availability

The aggregate counts of the phylum and genus level data (Supplement Materials Tables S1 and S2) and patient characteristics (Supplement Material Table S3) are partially available on the website, with the removal of any patient identifiers according to the IRB guidelines of Seoul St. Mary’s Hospital.

## Supplementary Materials

The following are available online at https://www.mdpi.com/article/10.3390/pathogens12010055/s1, Tables S1–S3.
